# Antifungal Susceptibility Patterns in Respiratory Tract Candidiasis and Risk Factors Among HIV-Infected Adults in South West Cameroon

**DOI:** 10.3390/antibiotics15090902

**Published:** 2026-09-14

**Authors:** Emile Valery Lyonga Bekende, Valantine Ngum Ndze, Erastus N. Nembo, Joseph Fokam, Taku Nadesh Ashukem, Nicoline Fri Tanih, Krisztián Bányai, Anna Longdoh Njunda

**Affiliations:** 1 Department of Medical Laboratory Science, Faculty of Health Sciences, University of Buea, Buea P.O. Box 63, Cameroon; lyvally@yahoo.com (E.V.L.B.); erastus.nembo@ubuea.cm (E.N.N.); josephfokam@gmail.com (J.F.); takuashukem@yahoo.com (T.N.A.); nicofriline@yahoo.com (N.F.T.); ann_njunda@yahoo.com (A.L.N.); 2 Department of Public Health and Hygiene, Faculty of Health Sciences, University of Buea, Buea P.O. Box 63, Cameroon; 3 Chantal BIYA International Reference Centre for Research on HIV/AIDS Prevention and Management, Yaoundé P.O. Box 3077, Cameroon; 4 National AIDS Control Committee, Yaoundé P.O. Box 1459, Cameroon; 5 Department of Medical Biology, Medical School, University of Pécs, 7624 Pécs, Hungary; bkrota@hotmail.com; 6 Department of Pharmacology and Toxicology, University of Veterinary Medicine, 1078 Budapest, Hungary; 7 National Laboratory of Infectious Animal Diseases, Antimicrobial Resistance, Veterinary Public Health and Food Chain Safety, HUN-REN Veterinary Medical Research Institute, 1143 Budapest, Hungary

**Keywords:** respiratory tract, candidiasis, HIV, viral load, antifungal sensitivity

## Abstract

**Background**: Respiratory tract candidiasis, primarily caused by *Candida* species, is a prevalent opportunistic fungal infection in individuals with HIV/AIDS. A high HIV viral load is considered a significant risk factor for developing respiratory fungal infection. This study aimed to identify the yeast species isolated from sputum samples of HIV-positive and HIV-negative adults, determine their antifungal susceptibility patterns, and investigate associated risk factors. **Methods**: A hospital-based comparative cross-sectional study was conducted from March to May 2022. Sputum samples were collected from 190 HIV-positive and 190 HIV-negative participants. *Candida* and other yeast species were isolated and identified, followed by antifungal sensitivity testing. **Results**: Of the 190 specimens from each group, 77 (40.53%) from HIV patients and 12 (6.31%) from HIV-negative participants tested positive for yeasts. *Candida albicans* was the most prevalent species, found in 52 (67.53%) HIV-positive patients and 12 (100%) HIV-negative patients. This was followed by *Nakaseomyces glabratus* (15.58%), *Pichia kudriavzevii* (9.09%), *Candida tropicalis* (6.49%), and *Candida parapsilosis* (1.29%) in HIV-positive patients. Notably, all 12 isolates from HIV-negative participants demonstrated 100% sensitivity to all ten tested antifungal drugs. In contrast, isolates from HIV patients showed significant resistance to fluconazole and ketoconazole (32.5% each). High viral load (*p* < 0.001), dermatophyte infection (*p* < 0.001), and antiretroviral (ARV) regimen (*p* = 0.030) were significantly associated with respiratory tract fungal infections in HIV patients. **Conclusions**: Based on this exploratory study, respiratory mycosis is frequent in HIV-positive patients in Cameroon, and identified fungi show increased resistance to azoles. While these results must be interpreted with care due to the sample size relative to the high national HIV prevalence, they suggest that public health education is needed to improve awareness. These preliminary findings prompt a review by health authorities concerning the use of fluconazole for prophylaxis in HIV-positive patients in Cameroon.

## 1. Introduction

Fungal respiratory tract infections are a major cause of morbidity and mortality among people living with human immunodeficiency virus (PLHIV), accounting for up to 70% of illnesses in acquired immune deficiency syndrome (AIDS) cases [1]. Concurrently, the global burden of invasive fungal infections (IFIs) has shown an upsurge in recent years, driven by a growing population of immunocompromised patients with various underlying medical conditions. Encompassed within this trend are opportunistic respiratory mycoses, a globally distributed, large group of fungal diseases whose etiological agents typically target immunocompromised or debilitated patients [2]. *Candida* and other yeast species serve as classic examples of these opportunistic pathogenic fungi.

Recently, significant advancements have been made in understanding the pathogenicity of candidiasis, including mechanisms of host tissue adherence, tissue penetration, intracellular multiplication, and fungal cell interaction with host effector cells [3]. Fungal pneumonia is typically observed as part of a disseminated infection, often associated with predisposing clinical conditions such as prolonged antibiotic use, hematologic malignancy, or severe immunosuppression [1,4]. Most fungal pneumonia cases result from hematological dissemination of *Candida* spp. from a distant site, commonly the gastrointestinal tract or skin [4]. Furthermore, the prevalence of other fungal co-infections, such as dermatophyte infections, has been noted as a potential indicator of severe immune depletion and a predisposing factor for respiratory *Candida* colonization in this population [5,6,7]. The clinical presentation can range from asymptomatic mucosal candidiasis to overwhelming disseminated infections. In PLHIV, fungal dissemination can lead to severe outcomes, highlighting the importance of understanding respiratory mycoses for early treatment.

In Cameroon, where the national HIV prevalence remains a critical challenge, the impact of opportunistic fungal infections is substantial, often complicating the clinical course for PLHIV [8,9,10]. Cameroon has a growing population of patients with AIDS and various immune deficiencies. Since opportunistic mycoses (candidiasis) pose a serious threat to such patients, these infections are anticipated to become a public health concern under suitable circumstances. The situation of HIV/AIDS in Cameroon is characterized by increasing antiretroviral therapy (ART) coverage; however, comprehensive resistance data remain sparse [11]. While first-line regimens are widely used, second-line ARV regimens involving protease inhibitors have shown the capacity to inhibit fungal secretory aspartyl proteinases, potentially influencing *Candida* prevalence and resistance patterns [12]. Conversely, the frequent use of fluconazole for prophylaxis without routine susceptibility testing has raised concerns about emerging resistance in the region.

The role of early and accurate diagnosis in the aggressive containment of the fungal infection at the initial stages becomes crucial as it can prevent the development of a life-threatening situation [13]. Clinical manifestations of invasive *Candida* infection are non-specific, and survival of HIV patients from such life-threatening infections depends on early diagnosis and appropriate treatment [14]. The emergence of these unusual infections has necessitated a re-evaluation of diagnostic tests for fungal infections and has profoundly influenced treatment choices [15]. In resource-limited settings such as Cameroon, early and accurate diagnosis of fungal infections is critical for the prevention of life-threatening clinical outcomes. However, the implementation of gold-standard molecular diagnostics, such as PCR or MALDI-ToF, remains severely constrained by the existing healthcare infrastructure. This lack of diagnostic capacity often necessitates the routine administration of antifungal prophylaxis without prior susceptibility testing, a practice that has raised significant concerns regarding emerging resistance in the region. Consequently, there is an urgent need for rapid, cost-effective, and standardized diagnostic platforms that can simultaneously perform species identification and antifungal profiling (examples include the Liofilchem^®^ Integral System Yeasts Plus (Liofilchem Diagnostici S.r.l, Roseto degli Abruzzi (TE), Italy; [https://www.liofilchem.net/login/pd/pi/71822_PI.pdf] (accessed on 16 January 2026)). Integrating these accessible diagnostic methods into routine clinical practice is essential not only for optimizing individual patient treatment for PLHIV but also for establishing robust antimicrobial resistance surveillance to safeguard public health.

The incidence and prevalence of respiratory fungal infections in Cameroon and many other developing countries remain largely unexplored and neglected [16]. Given its devastating effects, similar to other respiratory infections, this neglect is a major concern. Furthermore, in Fako Division, Cameroon, limited or no research has been conducted on respiratory tract fungal infections. Therefore, this study aimed to compare the prevalence of respiratory tract yeast infections in HIV-positive and HIV-negative adults, determine in vitro antifungal susceptibility patterns, assess the association between respiratory tract fungal infection and viral load in HIV-infected adults, and identify risk factors, including dermatophyte infections and specific ARV regimens, associated with respiratory tract mycoses in HIV-positive adults.

## 2. Results

### 2.1. Sociodemographic Data of Participants

A total of 380 participants were recruited for this study, comprising 190 HIV-positive and 190 HIV-negative adults. The mean ages were 47.51 ± 9.43 years for HIV-positive participants and 47.21 ± 9.43 years for HIV-negative participants. The majority of participants were in the age group of 46.0–55.0 years. Females constituted the dominant gender, with 126 (66.3%) for HIV-positive and 111 (58.4%) for HIV-negative participants. All study participants had achieved some level of education, with over half having completed primary education. Approximately half of the HIV-positive participants (82, 43.2%) were married, while the majority of HIV-negative participants (107, 56.3%) were married. There was a statistically significant association between HIV status and marital status (χ^2^ = 13.852, *p* = 0.001). Immunosuppressant use was also significantly associated with HIV status (χ^2^ = 26.850, *p* < 0.001). However, age group, gender, and level of education were not significantly associated with HIV status (Table 1).

### 2.2. Prevalence of Respiratory Tract Fungal Infections According to HIV Status

Among the 190 HIV-infected participants, 77 (40.53%) were positive for respiratory tract fungal infection. In contrast, the prevalence rate of respiratory mycoses among HIV-negative subjects was 6.31% (n = 12). Based on Fisher’s exact test, the highest prevalence rate of infection was observed in HIV-infected cases compared to non-HIV-infected cases, with a significant difference (*p* < 0.001).

### 2.3. Frequency of Isolated Yeast Species in Study Population

**Figure 1:**
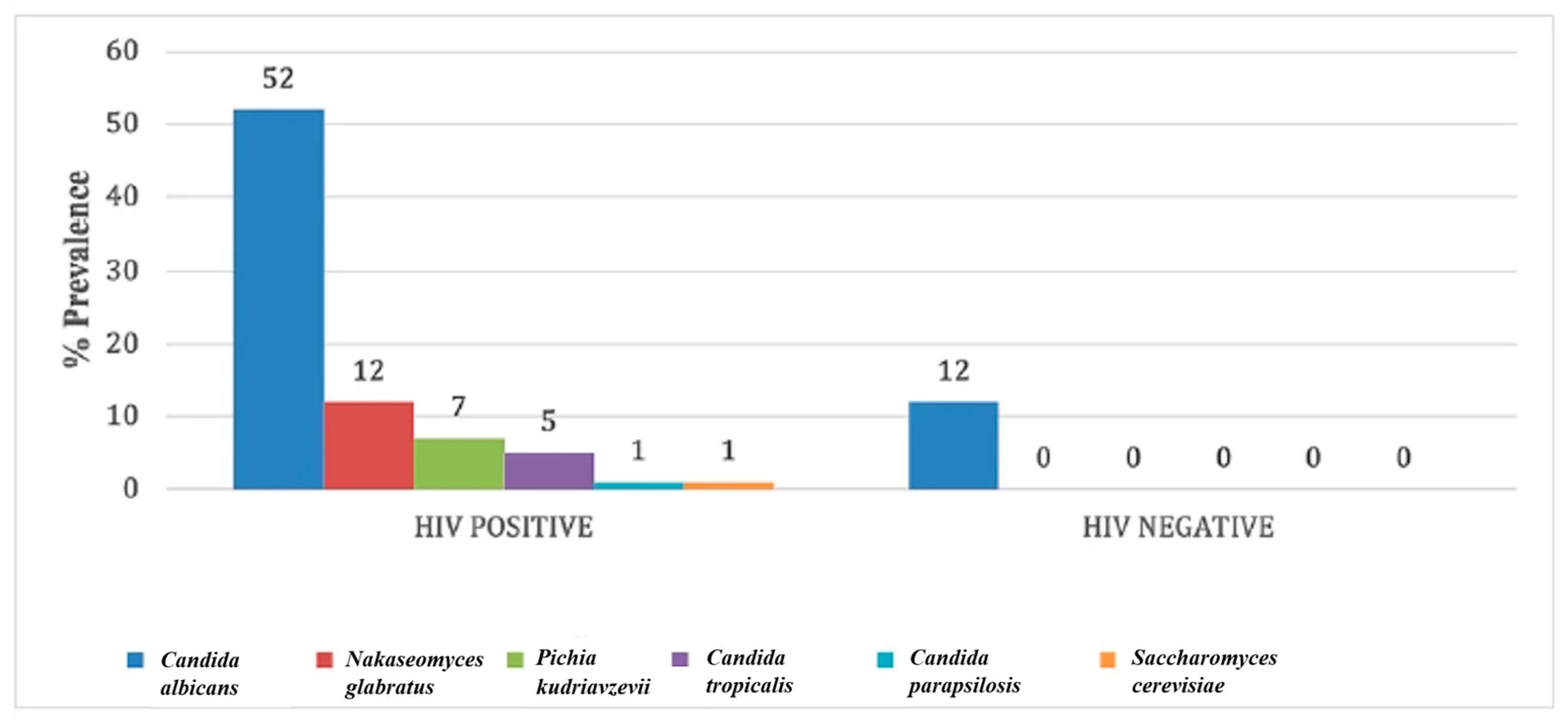
Frequency of the isolated yeast species based on HIV status.

Of the total 89 samples yielding yeast species growth (all single growth), 86.5% (77/89) were isolated from HIV-positive subjects and 13.5% (12/89) from HIV-negative subjects. Among the 77 samples from HIV-positive subjects, *Candida albicans* had the highest frequency, with 52 (67.53%) isolates, compared to non-albicans species. The non-albicans species and their frequencies included *Nakaseomyces glabratus* 12 (15.58%), *Pichia kudriavzevii* 7 (9.09%), *Candida tropicalis* 5 (6.49%), *Candida parapsilosis* 1 (1.29%) and *Saccharomyces cerevisiae* (1.29%; this case was not further characterized). All 12 samples that yielded yeast growth among the HIV-negative participants, were *C. albicans* (Figure 1).

### 2.4. Antifungal Susceptibility Pattern of Respiratory Yeast Isolates from Non-HIV Participants

All 12 isolates from HIV-negative participants were *C. albicans*, and 100% sensitivity was observed for all ten antifungal drugs tested.

### 2.5. Antifungal Susceptibility Pattern of Respiratory Yeast Species Isolated from HIV-Positive Patients

The sensitivity of the isolated fungal species ranged from 85.7% to 59.7%, with the isolates being most sensitive to nystatin (85.7%), followed by clotrimazole (75.3%), amphotericin B (74.0%), and miconazole (74.0%). The isolates demonstrated the highest rates of resistance to ketoconazole (32.5%) and fluconazole (32.5%), followed by econazole (27.3%) (Table 2).

### 2.6. Overall Antifungal Susceptibility Profile of Isolated Yeast Species

**Figure 2:**
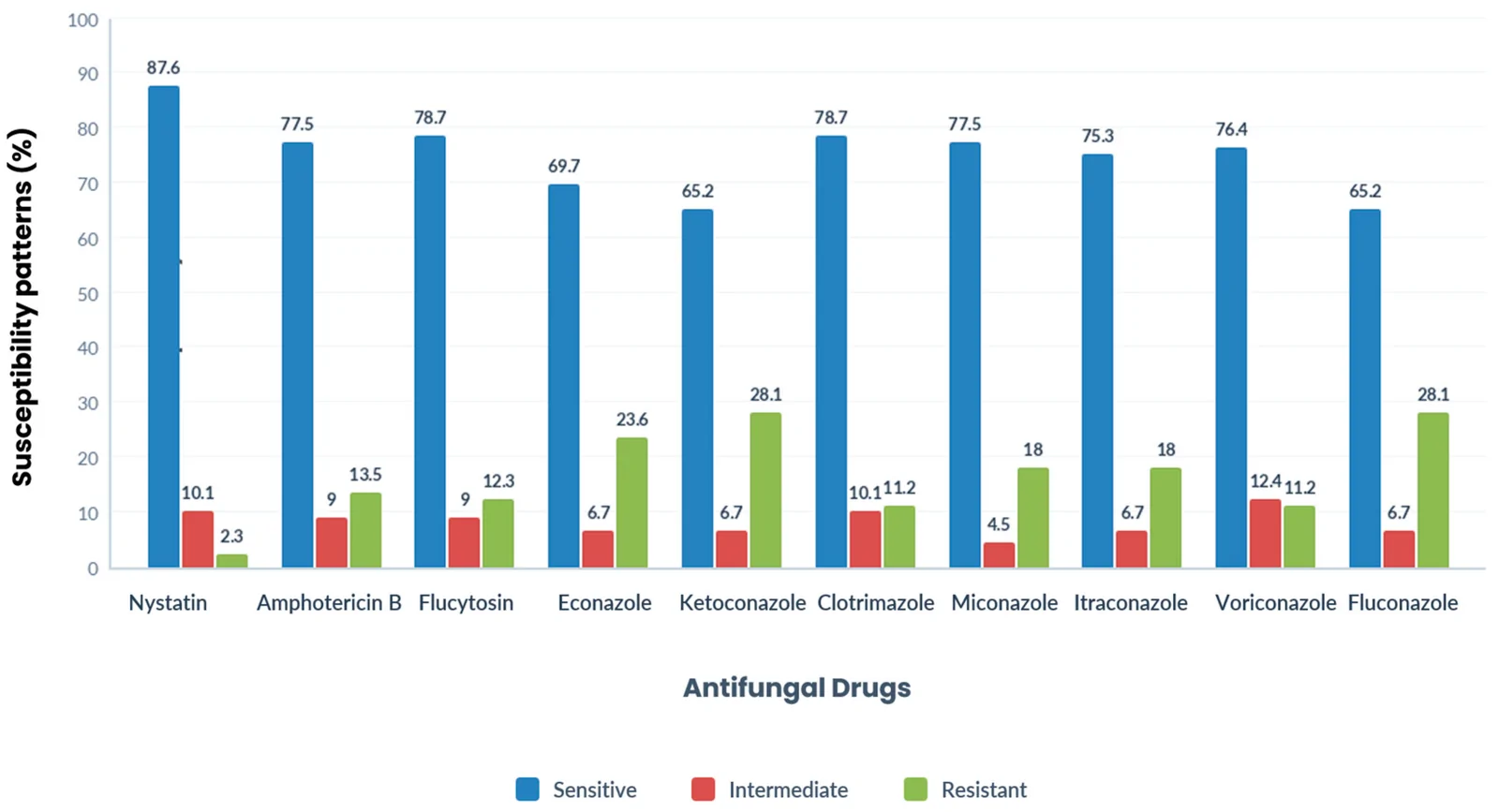
Overall antifungal susceptibility profile of isolated fungal species. The concentrations of antifungals were as follows: nystatin (1.25 µg/mL), amphotericin B (2 µg/mL), flucytosine (16 µg/mL), econazole (2 µg/mL), ketoconazole (0.5 µg/mL), clotrimazole (1 µg/mL), miconazole (2 µg/mL), itraconazole (1 µg/mL), voriconazole (2 µg/mL), and fluconazole (64 µg/mL).

Antifungal susceptibility was determined for all 89 fungal isolates obtained from positive cultures. The susceptibility of these isolates ranged from 87.6% to 65.2%. The isolates demonstrated the highest sensitivity to nystatin (87.6%), followed by flucytosine (78.7%), clotrimazole (78.7%), and amphotericin B (74.0%). In contrast, the isolates showed the highest levels of resistance to ketoconazole (28.1%) and fluconazole (28.1%), followed by econazole (23.6%) (Figure 2).

### 2.7. Association Between Respiratory Tract Fungal Infection and Predisposing Factors in HIV-Positive Patients

The highest prevalence rate (48.4%) was observed among patients aged over 55 years, while the lowest (30.8%) was in those under 35 years. However, there was no significant association between age group and respiratory fungal infection (*p* = 0.159). For gender, the higher prevalence rate (43.7%) was among females, but gender showed no significant association with respiratory fungal infection (*p* = 0.216). Regarding immunosuppressants, a higher prevalence rate (41.4%) was observed in those not on any immunosuppressant, while those on immunosuppressants had a prevalence rate of 38.6%. Immunosuppressants were not significantly associated with respiratory fungal infections (*p* = 0.750). A higher prevalence rate (42.5%) was seen in those on first-line antiretroviral (ARV) regimens compared to those on second-line ARV regimens (9.1%). ARV regimen showed a significant association with respiratory tract fungal infection (*p* = 0.030). For viral load, the highest prevalence rate (90.3%) was observed in those with viral loads between 41–1000 copies/mL, while the lowest (23.9%) was in those with viral loads less than 40 copies/mL. Viral load showed a significant association with respiratory tract fungal infection (*p* < 0.001). Dermatophyte infection also showed a significant association with respiratory fungal infection (*p* < 0.001), with a higher prevalence rate of 81.0% in those with positive dermatophyte infection (Table 3).

### 2.8. Association Between Respiratory Tract Fungal Infection and Predisposing Factors in HIV-Negative Participants

The highest prevalence rate (48.4%) was observed among participants older than 55 years, while the lowest prevalence rate of 30.8% was observed in those within the age group less than 35 years. Age showed no significant association with respiratory fungal infection (*p* = 0.353). Regarding gender, a higher prevalence rate (43.7%) was obtained among female participants, while the lowest (3%) was recorded in male participants. However, there was no significant association between gender and respiratory tract fungal infection (*p* = 0.530). In relation to immunosuppressants, a higher prevalence rate of 41.2% was obtained from those on immunosuppressants. A significant association was observed between respiratory fungal infection and immunosuppressant use (*p* < 0.001). A higher prevalence rate of 22.2% was observed in those positive for dermatophyte infection, and a significant association was found between dermatophyte infection and respiratory fungal infection (*p* = 0.012) (Table 4).

### 2.9. Risk Factors Associated with Respiratory Tract Fungal Infection in HIV-Positive Patients

Logistic regression analysis was used to estimate the OR of respiratory tract fungal infection as outcome variable. In the bivariate analysis, ARV regimens and viral load levels were significantly associated with respiratory tract fungal infections in HIV-positive patients. All variables with a *p* < 0.2 were included in the multiple logistic regression analysis (multivariate analysis). After adjusting for confounders in the multivariate analysis, ARV regimen, dermatophyte infections, and viral load level in HIV-positive patients were significantly associated with respiratory tract fungal infection. The odds of having respiratory tract fungal infections were significantly higher in HIV-positive patients presenting with dermatophyte infections (adjusted odds ratio (AOR): 2.15; 95% confidence interval (CI): 1.32–3.50, *p* < 0.001) compared to HIV patients without dermatophyte infections. Also, the odds of having respiratory tract fungal infections were significantly higher in those with a viral load of <1000 copies/mL (AOR: 0.08; 95% CI: 0.04–0.20; *p* < 0.001) compared to those with viral load >1000 copies/mL (Table 5).

## 3. Discussion

Respiratory tract *Candida* infection is among the most common opportunistic fungal infections in immunocompromised individuals [17]. Our study revealed that 40.53% of sputum samples from HIV-positive patients contained yeast colonies. This finding aligns with a similar study in Belém, Brazil, which reported a prevalence of 41.87% [18]. Other studies in Brazil and Cameroon reported higher prevalences of 60% and 54.1% respectively, possibly due to the fact that not all participants in those studies were on a first-line ARV regimen [19,20]. In HIV-negative participants, 6.31% of sputum samples showed positive growth for *Candida*, with all isolates identified as *C. albicans*. This contrasts with a study in Southwestern Uganda where *C. albicans* were the most isolated fungi amongst other types of fungi [21].

*C. albicans* was the most frequent species identified from both HIV-positive and HIV-negative participants in this study, consistent with previous studies in sub-Saharan Africa [18,21] and a study in Kuwait [22]. Similarly among HIV-positive patients, *C. albicans* was the predominantly isolated *Candida* species (67.52%) in this study as also observed in studies carried out in Brazil and Nigeria [19,23]. Amongst the HIV-negative patients, 100% of the *Candida* isolates were *C. albicans* which contrasts with a similar study in Nigeria [23]. The high prevalence of *C. albicans* in this study is likely facilitated by its virulence factors, particularly its multiple adhesins for host tissue adherence, biofilm formation, and secretion of hydrolytic enzymes like proteases, phospholipases, and hemolysins [24,25]. Beyond these factors, the dominance of *C. albicans* is further attributed to its superior ecological fitness and evolutionary adaptation as a primary human fungal commensal. Unlike many non-albicans species, *C. albicans* exhibits remarkable metabolic flexibility, allowing it to rapidly adapt to nutrient-poor environments and pH fluctuations within the host. This stability within the indigenous microbiota provides a competitive advantage, facilitating its rapid transition to an opportunistic pathogen when the host’s immune system is compromised. Additionally, *C. albicans* reversibly converts between unicellular yeast cells and pseudo hyphal or hyphal growth, a morphogenesis phenomenon where hyphal growth plays a crucial role in tissue invasion and resistance to phagocytosis [24,25]. Non-albicans species isolated from HIV-positive patients accounted for 32.47% of *Candida* species from the respiratory tract. This result aligns with a prevalence study of 44% reported in Brazil [23]. Among non-albicans species, *N. glabratus* (15.58%) was the most frequent, followed by *P. kudriavzevii* (9.09%), *C. tropicalis* (6.49%), and *C. parapsilosis* (1.29%). This finding is similar to other studies but shows some differences in percentages and species spectrum [17,23]. *N. glabratus*, *P. kudriavzevii*, and *C. tropicalis* were the most frequent non-albicans species isolated from HIV-positive patients, consistent with a study conducted in sub-Saharan Africa [17].

In this study, the prevalence of respiratory tract fungal infection was not found to be gender- or age-dependent. The carriage rate of fungal species for HIV-positive patients was higher in women (71.42%) than in men (28.57%), but this difference in prevalence was not statistically significant (*p* = 0.369). Yeast species prevalence in both HIV-positive and HIV-negative participants in the 46–55 years age group accounted for the highest prevalence rates (48.05% and 58.33%, respectively), but the difference in prevalence rates among age groups was not statistically significant (*p* = 0.159 and *p* = 0.353, respectively). This result aligns with studies conducted in Cameroon but differs from another study in Mexico, which showed age dependence on *Candida* colonization [20,26].

Importantly, significant associations were identified regarding clinical factors. In the distribution of respiratory tract fungal infection prevalence across different viral load levels, it was more frequent in individuals with 41–1000 copies/mL of viral load. There was a statistically significant association between the prevalence of respiratory tract fungal infection and high viral load (*p* < 0.001). This difference may be related to the immunodeficiency of the immune system. The prevalence of respiratory tract yeast infection was lower (9.1%) in HIV patients under second-line ARV regimens with protease inhibitors than in patients under first-line ARV regimens without protease inhibitors (42.5%). This finding is consistent with a study in Nigeria [27], and the prevalence of respiratory fungal infection was statistically significant (*p* = 0.029). This may be due to the fact that protease inhibitor groups of ARV drugs exert an early immune reconstitution-independent beneficial effect against respiratory *Candida* infections [28,29]. A low prevalence (4.1%) of respiratory tract fungal infection was observed in HIV patients with no dermatophyte infection, while a high prevalence (22.2%) was found in HIV patients with dermatophyte infections. This association was statistically significant (X^2^ = 56.837, *p* < 0.001).

In the bivariate analysis, dermatophyte infection, ARV regimens and viral load levels were significantly associated with respiratory tract fungal infections in HIV-positive patients. All variables with a *p*-value < 0.2 were considered for multivariate analysis. After adjusting for confounders in the multivariate analysis, ARV regimen, dermatophyte infections, and viral load level in HIV-positive patients were significantly associated with respiratory tract fungal infection. The odds of having respiratory tract fungal infections were significantly higher in HIV-positive patients presenting with dermatophyte infections (AOR: 2.15; 95% CI: 1.32–3.50, *p* < 0.001) compared to HIV-positive patients without dermatophyte infections. Additionally, the odds of having respiratory tract fungal infections were significantly higher in those on the second-line ARV regimen (AOR: 2.04; 95% CI: 1.10–3.82; *p* = 0.029) compared to those on the first-line ARV regimen. This may be attributed to the protease inhibitor group of ARV drugs exerting an early immune reconstitution-independent beneficial effect against respiratory *Candida* infections, entirely due to the protease inhibitor’s capacity to inhibit Sap enzymes of the *Candida* secretory aspartyl proteinase (Sap) family, which belongs to the same family as HIV-protease [29].

Management of fungal infections varies substantially depending on the anatomical localization of the infection, the patient’s immune status, and the isolated species [30]. To understand the therapeutic differences among isolates from respiratory tract *Candida*-infected participants, their sensitivities were tested. The antifungal agents evaluated in this study target distinct physiological pathways within the fungal cell. Flucytosine functions as an antimetabolite; once transported into the cell, it interferes with pyrimidine metabolism, ultimately inhibiting fungal DNA and protein synthesis. In contrast, polyenes, including Nystatin and Amphotericin B, act by binding to ergosterol in the fungal cell membrane, which leads to the formation of trans-membrane pores, resulting in the leakage of intracellular components and cell death. Lastly, azoles such as Fluconazole, Ketoconazole, and Clotrimazole share a common mechanism of action: they inhibit the enzyme lanosterol 14α-demethylase, thereby blocking the synthesis of ergosterol, an essential component of the fungal cell membrane [31]. Understanding these mechanisms is essential for interpreting the varying sensitivity patterns observed, particularly the increased resistance to azoles in HIV-positive individuals.

The results showed that 85.7% fungal isolates from HIV-positive patients were sensitive to Nystatin followed by Clotrimazole (75.3%), Amphotericin B (74.0%) and Miconazole (74.0%). Our results partly align with a similar study carried out in Mexico where most of the *Candida* isolates were sensitive to Nystatin and Amphotericin [32]. However, the outcome differs from a study carried out in Cameroon where most of the *Candida* species (85.5%) were sensitive to Ketonazole [20]. This difference is likely due to decreased sensitivity of the isolated *Candida* species to azoles. Generally, *Candida* species are susceptible to both azoles and polyenes, but antifungal susceptibility varies significantly between *C. albicans* and non-albicans *Candida* spp. Among the five yeast species isolates, *C. albicans* was the most sensitive to all ten antifungals tested compared to non-*C. albicans*. Among the non-*C. albicans* species isolated, *P. kudriavzevii* and *C. tropicalis* were the most resistant species to all antifungals tested.

In contrast, all *C. albicans* isolated from HIV-negative participants were sensitive to all antifungals. The difference in susceptibility patterns between isolates from HIV-positive and HIV-negative participants may depend on the study population, duration of prophylaxis, and treatment of patients against invasive fungal infections in each region. Antifungal drug susceptibility patterns vary by region, and species-specific resistance rates play an important role in resistance surveillance. Consistent susceptibility testing and determination of susceptibility patterns for antifungal agents appear necessary in routine clinical laboratory diagnosis particularly for HIV-positive individuals and antimicrobial resistance surveillance.

This study indicated resistance to fluconazole in 29.9% of respiratory *Candida* isolates. This prevalence is higher than rates obtained in different areas in Cameroon but is consistent with the 24.0% prevalence recorded in Benin City, Nigeria [20,27]. Possible explanations for the differences in rates include methodological variations, drug usage, immune status, regional variations, and intrinsic resistance of the *Candida* species [27]. Fluconazole is typically considered the drug of choice for both treatment and prophylactic prevention of fungal infections in HIV-infected individuals in Africa, owing to its favorable pharmacokinetic profile, low toxicity, and availability [17]. Consequently, increased resistance to the drug poses a threat to the treatment of respiratory fungal infections in HIV-positive patients. Other azoles showed resistance similar to fluconazole, contrary to a previous study in Mexico [32]. This is possibly due to cross-resistance to fluconazole, as all azoles exert the same mechanism of action, and fluconazole was routinely administered to HIV-positive patients with clinical symptoms of candidiasis without prior antifungal susceptibility testing in these centers.

While this research provides important insights, several limitations must be considered when interpreting the findings. First, the study relied on sputum samples for the isolation of *Candida* species. Although participants were given specific instructions to provide deep-seated specimens, this method inherently carries a higher risk of oral flora contamination compared to more invasive diagnostic procedures like bronchoalveolar lavage. Second, the research employed a hospital-based cross-sectional design conducted over a limited four-month period from February to May 2022. This design provides a specific snapshot of the study population rather than an observation of long-term epidemiological trends. Third, the focus on a single treatment center in Tiko, located in the South West Region of Cameroon, may not fully capture the diversity of resistance patterns across the entire country. These results should be interpreted with care given the sample size relative to the high national prevalence of HIV. Fourth, we acknowledge that the Liofilchem^®^ Integral System Yeasts Plus, while commercially validated for clinical use, may differ from the CLSI reference broth microdilution method. Furthermore, while molecular methods like PCR or MALDI-ToF are the gold standards for species identification, their use was limited by the diagnostic infrastructure available in the study region. Nonetheless, the combination of biochemical testing and Germ Tube assays provides a reliable baseline for understanding the local epidemiology of respiratory candidiasis. Finally, direct comparisons with international data are made more complex by potential methodological variations. Differences in regional drug usage, local antimicrobial resistance surveillance, and the intrinsic resistance of specific *Candida* species further influence the variability of these results compared to other regions.

## 4. Materials and Methods

### 4.1. Study Design and Patients

A comparative hospital-based cross-sectional study was designed which lasted for four months, from February to May 2022.

The study was conducted at Cottage Hospital, Tiko, a city approximately 18 km from Buea, the capital of the Southwest Region of Cameroon. Tiko has an estimated population of 334,647 people and a total surface area of 4840 km^2^, with an average population density of 28 inhabitants per square kilometer. Tiko is bordered to the west by Limbe, north by Buea, east by Dibombari, and south by Bonaberi. The hospital offers specialized services, including pediatrics, surgery, laboratory, maternity, medicine, ophthalmology, an HIV treatment center, and a pharmacy. The Cottage Hospital HIV treatment center manages over 5000 PLHIV, with an average of 30 HIV-positive patients visiting daily for ARV refills from various divisions of the Southwest Region. The hospital attends to an average of 3000 patients per month at the outpatient department. The laboratory has a well-equipped microbiology unit with all necessary equipment for effective collection and analysis of sputum samples for *Candida* species.

The study population included all adults aged 21 years and above, both HIV-negative and HIV-positive. Participants were selected consecutively as they visited the hospital. Dependent variables included fungal species infection and antifungal susceptibility patterns, while independent variables included age, sex, marital status, occupational status, educational level, clinical signs and symptoms of respiratory candidiasis, previous history of respiratory infections, and previous history of hospitalization.

Questionnaires were administered to prospective HIV participants during their visits to the HIV treatment center for ARV refill and blood collection for viral load monitoring. Questionnaires were administered to HIV-negative participants at the outpatient department of the hospital. For illiterate participants, questionnaires were read and explained to them.

### 4.2. Ethical Approval

Informed consent was obtained from participants who were involved in this study. This study was conducted according to the guidelines of the Declaration of Helsinki and approved by the Institutional Review Board of the Faculty of Health Sciences University of Buea, Cameroon (IRB # 1676-02).

### 4.3. Culture Media Preparation

Sixty-five grams (65.0 g) of Sabouraud Dextrose powder were measured using a light-sensitive balance, suspended in 1 L of distilled water, and heated under a Bunsen burner until completely dissolved. The preparation was autoclaved at 121 °C for 15 min. It was allowed to cool to approximately 30 °C, with 15 mL poured per Petri dish and preserved at 2–8 °C. Prior to media utilization, a sterility control was done.

### 4.4. Sample Collection

Specimen containers were labeled with serial numbers, patient codes, and dates. Participants were instructed to collect specimens in a well-ventilated area, away from other people. They were advised to take three deep breaths in and out, followed by a cough originating deep within the chest cavity. This sequence was repeated until an adequate amount of sputum (1 mL–3 mL) was collected. The specimen container was then closed and placed into the specimen reception bucket at the microbiology unit of the laboratory.

### 4.5. Sample Analysis

Sputum culture, germ tube test, species identification and antifungal testing were carried out.

Sputum samples were inoculated on Sabouraud Dextrose Agar (SDA) supplemented with Chloramphenicol and incubated at 35 °C (±2 °C) for 48 h under aerobic conditions. White to cream-colored, smooth, glabrous yeast-like colonies indicated yeast growth.

A germ tube test was performed for all positive growth cultures for presumptive identification of *C. albicans*. Approximately 0.5 mL of sterile human serum was dispensed into a test tube using a sterile wire loop. The serum was inoculated with 1–2 discrete colonies of the test organism. The test tube was incubated at 37 °C for 2–3 h. After incubation, a drop of the serum-yeast culture was transferred to a clean, grease-free glass slide and covered with a coverslip. It was then examined microscopically using ×10 and ×40 objectives with the diaphragm sufficiently closed to provide good contrast. Sprouting or tube-like outgrowths from the cells indicated hypha formation.

All yeast positive growth isolates from each patient were subjected to the Liofilchem^®^ Integral System Yeasts plus (ref. 71822) (Liofilchem Diagnostici S.r.l, Roseto degli Abruzzi (TE), Italy) for identifying different *Candida* species and performing antifungal susceptibility testing. For AST interpretation, it follows the principles of CLSI (Clinical and Laboratory Standards Institute) and EUCAST (European Committee on Antimicrobial Susceptibility Testing) even though the system is not a CLSI or EUCAST standard. This system consists of a single-use disposable plastic strip with 12 wells for 12 colorimetric biochemical tests for sugar assimilation, including glucose, maltose, sucrose, lactose, galactose, melibiose, cellobiose, inositol, xylose, raffinose, trehalose, and dulcitol. Well 13 contained a chromogenic media for differentiating *Candida* species based on color change. Wells 14 to 23 contained the following antifungals: nystatin (1.25 µg/mL), amphotericin (2 µg/mL), flucytosine (16 µg/mL), econazole (2 µg/mL), ketoconazole (0.5 µg/mL), clotrimazole (1 µg/mL), miconazole (2 µg/mL), itraconazole (1 µg/mL), voriconazole (2 µg/mL), and fluconazole (64 µg/mL). Well 24 served as a growth control. Inoculation of wells 01 to 13 involved adding 0.2 mL of a yeast suspension (0.5 McFarland standard) to the dehydrated substrates. From the 0.5 McFarland standard turbidity preparation, 0.02 mL was transferred into 4.5 mL of physiological solution, which was used to inoculate wells 14 to 24. A drop of oil was then placed on the surface of each well, except well 13. Results were read after incubation for 48 h at 36 °C ± 1. In wells 01 to 12, a color change from yellow to grey signified positive sugar assimilation, while a purple color indicated negative sugar assimilation. A four-digit numerical profile was generated for each isolate based on the reactions produced. Fungal species identifications were made by referring to a list of numerical profiles. At well 13, specific colors indicated the presence of the following species: *C. albicans* (green), *N. glabratus* (purple), *P. kudriavzevii* (pink), *C. parapsilosis* (cream), *C. tropicalis* (blue), and *C. pseudotropicalis* (white or mauve). Susceptibility in wells 14 to 23 was interpreted based on color presentation: red, signified sensitive; orange, intermediate; yellow, resistant [33].

For HIV viral load (VL) measurement, each participant whole blood sample was collected in EDTA tubes by trained healthcare workers, centrifuged and plasma specimens aliquoted at various collection sites (spokes), stored in cold chain (−20 °C or −80 °C) and then shipped to the reference laboratory in Limbe for plasma VL analysis. Briefly, plasma samples extracted, RNA was reverse-transcribed and then amplified using a Real-Time PCR as per the manufacturer’s instructions: Gene Expert point-of-care technology of Cepheid (limit of detection, LoD < 40 copies/mL); Abbott Real-Time m2000 system (LoD < 40 copies/mL); and open platform of Biocentric (LoD < 390 copies/mL).

### 4.6. Data Management

Data were recorded on register forms and entered into a Microsoft Excel database on a secure computer. Analysis was performed using SPSS version 20 and EPI info version 7. Data were statistically described using frequencies and percentages. The *p*-value for dichotomous variables such as gender, immunosuppressants, dermatophyte infection, and being under ARV regimen was obtained using Fisher’s exact test. Multiple logistic regression analysis was applied to analyze risk factors associated with respiratory tract candidiasis. A *p*-value less than 0.05 was considered statistically significant at a 95% confidence interval.

## 5. Conclusions

This study underscores the significant burden of respiratory tract fungal (mainly *Candida*) infections among HIV-positive adults in South West Cameroon, revealing a prevalence (40.5%) nearly seven times higher than that of HIV-negative individuals. These findings have profound implications for clinical practice and public health policy in the region. The high frequency of infection in patients with elevated viral loads and those on first-line ARV regimens highlights a critical window for targeted screening. Furthermore, the emergence of non-albicans species, such as *P. kudriavzevii*, *C. tropicalis*, and *N. glabratus*, which demonstrated the highest rates of multi-drug resistance, presents a major diagnostic challenge. This shift in the mycological profile suggests that clinical management can no longer rely on the assumption of *C. albicans* dominance; instead, species-level identification is becoming essential for effective treatment. Perhaps the most significant finding is the 28.1% resistance rate to fluconazole across all isolates. Given that fluconazole remains the primary agent for both prophylaxis and treatment of fungal infections in sub-Saharan Africa due to its availability and low cost, this level of resistance poses a direct threat to patient outcomes. Our results suggest an urgent need for health authorities in Cameroon to re-evaluate current prophylactic protocols and prioritize the implementation of routine antifungal susceptibility testing in clinical laboratories. Finally, the strong association between respiratory candidiasis and factors such as dermatophyte co-infections and ARV regimen types provides clinicians with practical markers to identify high-risk patients. By integrating these preliminary findings into public health education and HIV management strategies, healthcare providers can improve early detection and containment of potentially life-threatening invasive fungal infections. This preliminary investigation serves as a necessary baseline for broader antimicrobial resistance surveillance and the optimization of opportunistic infection management in Cameroon.

## Figures and Tables

**Table 1:** Sociodemographic data of participants.

Variables	HIV Patients		HIV Negative		Total		
	Frequency (n)	%	Frequency (n)	%	Frequency (n)	%	*p*-Value
**Age Group (years)**							
≤35	13	6.8	13	6.8	26	6.8	0.991
36–45	70	36.8	72	37.8	142	37.4	
46–55	76	40.0	73	38.4	149	39.2	
>55	31	16.3	32	16.8	63	16.6	
TOTAL	190	100	190	100	380	100	
**Gender**							
Female	126	66.3	111	58.4	237	62.4	0.112
Male	64	33.7	79	41.6	143	37.6	
TOTAL	190	100	190	100	380	100	
**Marital Status**							
Married	82	43.2	107	56.3	189	49.7	0.001
Single	81	42.6	75	39.5	156	41.1	
Widow	27	14.2	8	4.2	35	9.2	
TOTAL	190	100	190	100	380	100	
**Level of education**							
Primary	123	64.7	122	64.2	245	64.5	1.000 *
Secondary	64	33.7	65	34.2	129	33.9	
Tertiary	3	1.6	3	1.6	6	1.6	
TOTAL	190	100	190	100	380	100	
**Immunosuppressants**							
No	133	70.0	173	91.1	306	80.5	<0.001
Yes	57	30.0	17	8.9	74	19.5	
TOTAL	190	100	190	100	380	100	

* Fisher exact test was performed.

**Table 2:** Antifungal susceptibility pattern of respiratory fungal species isolated in HIV-positive patients.

Fungal Species		NYS n (%)	AMB n (%)	FCY n (%)	ECN n (%)	KCA n (%)	CLO n (%)	MIC n (%)	ITR n (%)	VOR n (%)	FLU n (%)
***C. albicans***	S	46 (88.5)	43 (82.7)	42 (80.8)	36 (69.2)	34 (65.4)	43 (82.7)	42 (80.8)	38 (73.1)	38 (73.1)	34 (65.4)
**(n = 52)**	I	1 (1.9)	4 (7.7)	5 (9.6)	3 (5.8)	4 (7.7)	4 (7.7)	2 (3.8)	4 (7.7)	5 (9.6)	3 (5.8)
	R	5 (9.6)	5 (9.6)	5 (9.6)	13 (25.0)	14 (26.9)	5 (9.6)	8 (15.4)	10 (19.2)	9 (17.3)	15 (28.8)
***N. glabratus***	S	10 (83.3)	8 (66.7)	8 (66.7)	6 (50.0)	5 (41.7)	7 (58.3)	6 (50.0)	9 (75.0)	9 (75.0)	6 (50.0)
**(n = 12)**	I	0 (0.0)	1 (8.3)	0 (0.0)	2 (16.7)	2 (16.7)	1 (8.3)	2 (16.7)	1 (8.3)	2 (16.7)	1 (8.3)
	R	2 (16.7)	3 (25.0)	4 (33.3)	4 (33.3)	5 (41.7)	4 (33.3)	4 (33.3)	2 (16.7)	1 (8.3)	5 (41.7)
***P. kudriavzevii***	S	5 (71.4)	3 (42.9)	4 (57.1)	4 (57.1)	3 (42.9)	4 (57.1)	4 (57.1)	4 (57.1)	5 (71.4)	3 (42.8)
**(n = 7)**	I	0 (0.0)	2 (28.6)	2 (28.6)	1 (14.3)	0 (0.0)	1 (14.3)	0 (0.0)	1 (14.3)	2 (28.6)	1 (14.3)
	R	2 (28.6)	2 (28.6)	1 (14.3)	2 (28.6)	4 (57.1)	2 (28.6)	3 (42.8)	2 (28.6)	0 (0.0)	3 (42.8)
***C. tropicalis***	S	4 (80.0)	2 (40.0)	3 (60.0)	3 (60.0)	3 (60.0)	4 (80.0)	4 (80.0)	3 (60.0)	3 (60.0)	2 (40.0)
**(n = 5)**	I	1 (20.0)	1 (20.0)	1 (20.0)	0 (0.0)	0 (0.0)	1 (20.0)	0 (0.0)	0 (0.0)	2 (40.0)	1 (20.0)
	R	0 (0.0)	2 (40.0)	1 (20.0)	2 (40.0)	2 (40.0)	0 (0.0)	1 (20.0)	2 (40.0)	0 (0.0)	2 (40.0)
***C. parapsilosis***	S	1 (100)	1 (100)	1 (100)	1 (100)	1 (100)	1 (100)	1 (100)	1 (100)	1 (100)	1 (100)
**(n = 1)**	I	0 (0.0)	0 (0.0)	0 (0.0)	0 (0.0)	0 (0.0)	0 (0.0)	0 (0.0)	0 (0.0)	0 (0.0)	0 (0.0)
	R	0 (0.0)	0 (0.0)	0 (0.0)	0 (0.0)	0 (0.0)	0 (0.0)	0 (0.0)	0 (0.0)	0 (0.0)	0 (0.0)
**Total**	S	66 (85.7)	57 (74.0)	58 (73.3)	50 (64.9)	46 (59.7)	58 (75.3)	57 (74.0)	55 (71.4)	56 (72.7)	46 (59.7)
**(n = 77)**	I	9 (11.7)	8 (10.4)	8 (10.4)	6 (7.8)	6 (7.8)	9 (11.7)	4 (5.2)	6 (7.8)	11 (14.3)	6 (7.8)
	R	2 (2.6)	12 (15.7)	11 (14.3)	21 (27.3)	25 (32.5)	10 (13.0)	16 (20.8)	16 (20.8)	10 (13.0)	25 (32.5)

NYS = Nystatin, AMB = Amphotericin B, FCY = Flucytosine, ECN = Econazole, KCA = Ketoconazole, CLO = Clotrimazole, MIC = Miconazole, ITR = Itraconazole, VOR = Voriconazole, FLU = Fluconazole; S = Sensitive, I = Intermediate, R = Resistant.

**Table 3:** Association between respiratory yeast infection and predisposing factors in HIV patients.

Variables	Number Examined	Negative (%)	Positive (%)	*p*-Value
**Age group (years)**				0.159
≤35	13	9 (69.2)	4 (30.8)	
36–45	70	48 (68.6)	22 (31.4)	
46–55	76	40 (52.6)	36 (47.4)	
>55	31	16 (51.6)	15 (48.4)	
**Gender**				0.216
Female	126	71 (56.3)	55 (43.7)	
Male	64	42 (65.6)	22 (34.4)	
**Immunosuppressants**				0.750
No	173	78 (58.6)	55 (41.4)	
Yes	17	35 (61.4)	22 (38.6)	
**Antiretroviral regimen**				0.030
First-line regimen	179	103 (57.5)	76 (42.5)	
Second-line regimen	11	10 (90.9)	1 (9.1)	
**Viral load(copies/mL)**				<0.001
≤40	142	108 (76.1)	34 (23.9)	
41–1000	31	3 (9.7)	28 (90.3)	
<1000	17	2 (11.8)	15 (88.2)	
**Dermatophyte infection**				<0.001
Negative	132	102 (77.3)	30 (22.7)	
Positive	58	11 (19.0)	47 (81.0)	

**Table 4:** Association between respiratory tract fungal infection and predisposing factors in HIV-negative participants.

Variables	Number Examined	Negative (%)	Positive (%)	*p*-Value
**Age group (years)**				0.353
≤35	13	9 (69.2)	4 (30.8)	
36–45	72	48 (68.6)	22 (31.4)	
46–55	73	40 (52.6)	36 (47.4)	
>55	32	16 (51.6)	15 (48.4)	
**Gender**				0.530
Female	111	106 (95.5)	5 (43.7)	
Male	79	73 (92.4)	6 (34.4)	
**Immunosuppressants**				<0.001
No	173	169 (97.7)	4 (2.3)	
Yes	17	10 (58.8)	7 (41.2)	
**Dermatophyte infection**				0.012
Negative	172	165 (95.9)	7 (4.1)	
Positive	18	14 (77.8)	4 (22.2)	

**Table 5:** Risk factors associated with respiratory tract fungal infection in HIV-positive patients.

Variables	COR (95% CI)	*p*-Value	AOR (95%CI)	*p*-Value
**Antiretroviral regimen**				
First line	1			
Second line	0.76 (0.46–1.28)	0.029	2.04 (1.10–3.82)	0.029
**Viral load (copies/mL)**				
≤40	0.09 (0.05–0.21)	<0.001	0.09 (0.04–0.19)	<0.001
41–1000	0.11 (0.05–0.24)	<0.001	0.08 (0.04–0.20)	<0.001
>1000	1		1	
**Dermatophyte infection**				
Negative	1		1	
Positive	1.92 (1.25–2.94)	<0.001	2.15 (1.32–3.50)	<0.001

**COR:** Crude Odd Ratio; **AOR:** Adjusted Odds Ratio.

## Data Availability

The clinical data presented in this study are available on request from the corresponding author. The data are not publicly available due to ethical restrictions.
